# Self-Assembled Hydrogel Nanoparticles for Drug Delivery Applications

**DOI:** 10.3390/ma3021420

**Published:** 2010-02-24

**Authors:** Catarina Gonçalves, Paula Pereira, Miguel Gama

**Affiliations:** IBB-Institute for Biotechnology and Bioengineering, Centre for Biological Engineering, Minho University, Campus de Gualtar 4710-057, Braga, Portugal; E-Mails: cgoncalves@deb.uminho.pt (C.G.); paulapereira@deb.uminho.pt (P.P.)

**Keywords:** polymer, delivery, targeting, self-assembly, drugs

## Abstract

Hydrogel nanoparticles—also referred to as polymeric nanogels or macromolecular micelles—are emerging as promising drug carriers for therapeutic applications. These nanostructures hold versatility and properties suitable for the delivery of bioactive molecules, namely of biopharmaceuticals. This article reviews the latest developments in the use of self-assembled polymeric nanogels for drug delivery applications, including small molecular weight drugs, proteins, peptides, oligosaccharides, vaccines and nucleic acids. The materials and techniques used in the development of self-assembling nanogels are also described.

## 1. Introduction

Nanotechnology is the source of exciting progresses in the drug delivery field, offering suitable means for site-specific and time-controlled delivery of small molecular weight drugs, proteins, peptides, oligosaccharides, vaccines and nucleic acids [1,2,3,4,5]. Overall, drug delivery is the method of administering a bioactive compound to achieve a therapeutic effect, in humans or animals. Drug delivery systems are formulations that modify the drug release profile and the ability to cross biological barriers, the biodistribution and pharmacokinetics, improving its efficacy and safety, as well as the patient compliance. Nanoformulations for drug delivery include numerous architectural designs in terms of size, shape, and materials. Several types of nanoparticles have been tested as potential drug delivery systems, including hydrogel nanoparticles (also known as polymeric nanogels or macromolecular micelles), dendrimers [6], nanospheres [7], nanocapsules and liposomes [8]. Each kind of formulation has characteristic drug loading capacity, particle and drug stability, drug release rates, and targeting ability. This review highlights the use of self-assembled hydrogel nanoparticles for drug delivery applications. Hydrogels are polymeric networks with three-dimensional configuration that absorb large quantities of water or biological fluids. Their water affinity is attributed to the presence of hydrophilic groups—such as ether, amine, hydroxyl, sulfate and carboxyl—in the polymer chains. Hydrogels can be formulated as macroscopic networks, or confined to smaller dimensions. When their size is in the submicron range, they are known as nanogels [9]. Nanogels, or hydrogel nanoparticles, have gained considerable attention as one of the most promising nanoparticulate drug delivery systems, owing to their unique properties that combine the characteristics of hydrogel systems (e.g., rather high water content) with a very small size (nanosize). Hydrogel nanoparticles are outstanding drug delivery systems:

1. The particle size and surface properties can be manipulated to avoid rapid clearance by phagocytic cells, allowing both passive and active drug targeting;

2. Controlled and sustained drug release at the target site, improving the therapeutic efficacy and reducing side effects. Drug loading is relatively high and may be achieved without chemical reactions; this is an important factor for preserving the drug activity;

3. Ability to reach the smallest capillary vessels, due to their tiny volume, and to penetrate the tissues either through the paracellular or the transcellular pathways;

4. Potential for administration through various routes, including oral, pulmonary, nasal, parenteral, intra-ocular *etc.*

This review paper is focused on the self-assembled hydrogel nanoparticles for biomedical applications, particularly drug delivery. Self-assembled hydrogel nanoparticles in this context refer to nanogels formed from amphiphilic or polyionic polymers. The main properties of nanoparticles, the method and materials for their production, and the main applications will be reviewed.

## 2. Materials, Properties, Methods

### 2.1. Materials

A wide range of materials may be used for nanogels preparation. Biodegradability is essential to avoid organ accumulation, potentially leading to toxicity and other undesirable side effects [10,11]. Hydrogel nanoparticles are made either from natural or synthetic polymers. The former possess a high variety of functional groups, which allow chemical and biochemical modification resulting in many kinds of biopolymer-based materials. Among these, polysaccharides are the more often used. Polysaccharides are naturally occurring carbohydrate-based polymers, formed of repeating units (monosaccharides) joined together by glycosidic bonds. They may be obtained from algal (alginate), plant (cellulose, starch) and animal (chitosan) sources, exhibit quite variable structures and properties, different reactive groups, a wide range of molecular weights and variable chemical composition. Polysaccharides can be divided into non-polyelectrolytes (dextran, dextrin, pullulan) and polyelectrolyte. Polyelectrolyte polysaccharides may be further divided into positively (chitosan) and negatively (heparin, hyaluronic acid) charged. Polysaccharides are highly stable, safe, non-toxic, hydrophilic and biodegradable. They are, in addition, abundant natural resources and may be processed at low cost.

Among the more often used synthetic polymers, block copolymers consist of two or more segments of simple polymers (blocks) joined in some arrangement. Block copolymers are further classified by the number of blocks each molecule contains: two, three, or more blocks correspond to diblocks, triblocks, or multiblocks, respectively. Biodegradable and biocompatible poly(d,l lactic acid) (PLA), poly(glycolic acid) (PGA) and their copolymer poly(d,l-lactic-co-glycolic acid) (PLGA) have been extensively used in controlled drug delivery and have been approved by the US Food and Drug Administration [12].

### 2.2. Properties

The ability of nanodelivery systems to overcome physiological barriers is determined by properties such as particle size, surface charge and hydrophobicity. Physiological barriers are biological structures or physiologic mechanisms that hinder nanoparticles from reaching their targets, therefore compromising the therapeutic efficacy. Nanoparticulate systems able to overcome these biological barriers should be capable of: efficient extravazation through the vasculature, prolonged vascular circulation time, improved cellular uptake and endosomal escape.

**Figure 1:**
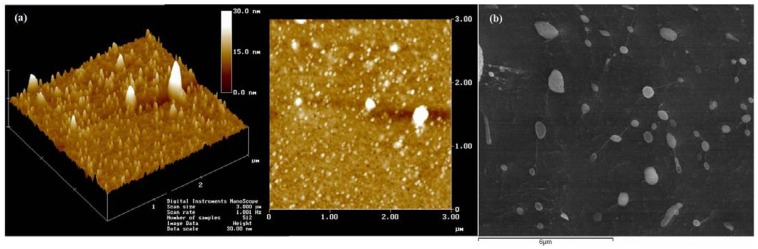
Size of dextrin nanoparticles evaluated by (a) atomic force microscopy and (b) cryo-scanning electron microscopy, adapted from [19].

The endothelial wall in the vasculature presents reduced permeability and constitutes the primary barrier for nanoparticles. The nanosize (Figure 1) may facilitate the penetration of nanoparticles across the endothelium. It is well known that extravazation of nanoparticles into the brain, across the blood-brain barrier, represents a particular difficult challenge for nanomedicine. Small drugs may diffuse through the capillary walls into the tissues. Otherwise, nanoparticles transport occurs through compromised endothelial barrier or mediated by specific transport systems. Tumor tissues have an increased capillary permeability, which allows a high rate of nanoparticles accumulation, based on the “enhanced permeability and retention” (EPR) effect [13]. Nanoparticles circulating in the bloodstream are normally able to penetrate the tissues through a paracellular path only at restricted sites, where the capillaries have open fenestrations, as in the sinus endothelium of the liver, in the tumor neovasculature [14] or when the endothelial barrier is altered by inflammatory processes (e.g., rheumatoid arthritis, infections) [15].

Surface charge is important as it determines the stability of nanoparticles, hence its propensity to aggregate in the bloodstream or interact with the cell membranes. The zeta potential is commonly used to characterize and measure the surface charge: the larger the absolute value of the zeta potential, the larger the charge on the surface. In a sense, the zeta potential represents an index for particle stability. For charged particles, as the zeta potential increases the repulsive interactions becomes larger, leading to stable particles, likely to have a more uniform size distribution. A nanosuspension stabilized by electrostatic repulsion must have a minimum zeta potential of ±30 mV [16]. This stability is important in preventing aggregation [17]. When a surface modifier like PEG is added, the negative zeta potential is lowered, although increasing the nanoparticles stability due to steric effects and hydration forces [18].

**Figure 2:**
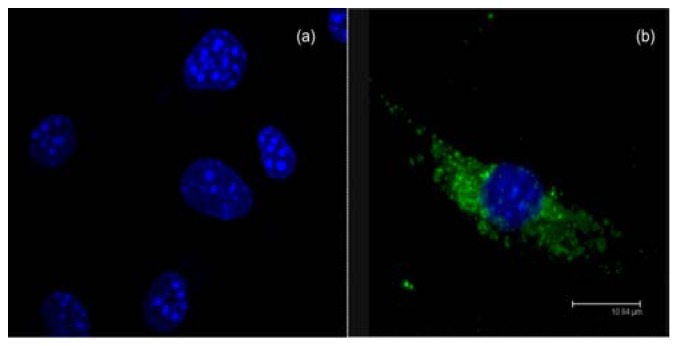
Fluorescence images obtained by confocal microscopy of murine bone marrow-derived macrophages (a) incubated without nanoparticles and (b) with FITC-containing nanoparticles (green). Nucleus stained with DAPI (blue), adapted from [19].

Long circulation time increases the odds for the nanoparticles to reach their target. The Mononuclear Phagocyte System (MPS) efficiently eliminates nanoparticles from the bloodstream, unless they are modeled to escape recognition. Opsonization is the process whereby a foreign organism or particle becomes covered with opsonins, thereby making it “visible” to phagocytic cells. This process typically occurs in the bloodstream and can take anywhere from a matter of seconds to many days to complete. Binding of opsonins onto the nanoparticle surface acts as a bridge between nanoparticles and phagocytes. Figure 2 shows bone marrow-derived macrophages with internalized polymeric nanoparticles labeled with FITC. Minimizing protein binding is the key point for developing long circulating time formulations. Together, these two processes—opsonization and phagocytosis—constitute the main blood clearance mechanism for particles larger than the renal threshold limit. There are no absolute rules or methods available to completely block opsonization, but research over the last 30 years yielded useful trends and methods to increase the blood circulation half-life and the effectiveness of “stealth” devices. For instance, it is well established that hydrophilic polymers provide steric stabilization and confers “stealth” invisibility for the body´s natural defense system [20]. As a general rule, the opsonization of hydrophobic particles, as compared to hydrophilic ones, has been shown to occur quicker, due the enhanced adsorption of blood serum proteins [21]. A correlation between surface charge and opsonization has also been demonstrated *in vitro*, neutral particles performing better than charged ones [22]. Therefore, a widely used method to slowdown opsonization relies on the use of shielding groups which can block electrostatic and hydrophobic interactions that help opsonins binding the nanoparticle surfaces. These groups are typically long hydrophilic polymer chains and non-ionic surfactants. Examples of effective shielding groups include polysaccharides, polyacrylamide, poly(vinyl alcohol), poly(*N-*vinyl-2-pyrrolidone), poly(ethylene glycol) (PEG), and PEG-containing copolymers such as poloxamers, poloxamines, polysorbates, and PEG copolymers [20,23]. Among the polymers tested to date, the most effective and most used are PEG and PEG-containing copolymers, typically very flexible, highly hydrophilic and not charged, altogether properties lessening the protein binding.

The high mobility of nanoparticles in the smallest capillaries allows for efficient uptake and selective drug accumulation at target sites [24]. Indeed, cell uptake of nanoparticles is relatively high, when compared to microparticles (>1 µm) [25]. Specific cell internalization can be improved by surface decoration with targeting ligands (peptide, sugar molecule, antibody, vitamins), which are recognized by target cells/tissues. The release of bioactive molecules into the cytoplasm or nucleous compartments can be a challenging problem. Internalized particles are initially within endosomes but are trafficked rapidly to lysosomes, where they are degraded enzymatically, preventing their action. To avoid lysosomal trafficking, smart polymers with specific chemical groups have been designed.

Although nanogels are indeed rather promising, it must be stressed that their development as products for clinical applications still requires further knowledge on its physico-chemical properties, knowledge on the interaction with cells and tissues, nanotoxicology and safety. Generally, nanoparticles that have a mean diameter of approximately 100 nm, bearing a neutral and hydrophilic surface, exhibit prolonged blood circulation and an increased level of tumor delivery [14].

### 2.3. Methods

**Figure 3:**
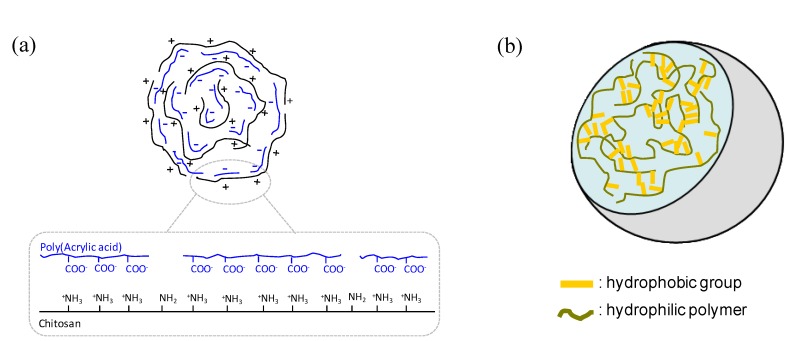
Schematic representation of intermolecular interactions driving self-assembly processes that includes (a) electrostatic interactions and (b) hydrophobic association.

The self-assembly process, defined as the autonomous organization of components into structurally well-defined aggregates, is characterized by numerous beneficial attributes; it is cost-effective, versatile and facile; the process occurs towards the system’s thermodynamic minima, resulting in stable and robust structures. Molecular self-assembly is characterized by diffusion followed by specific association of molecules through non-covalent interactions, including electrostatic and/or hydrophobic associations (Figure 3). Individually, such interactions are weak, but dominate the structural and conformational behavior of the assembly due to the large number of interactions involved [26]. While oppositely charged polysaccharides associate readily as a result of electrostatic attractions [27], interactions among neutral polysaccharides tend to be weaker, or nonexistent, a modification with chemical entities able to trigger assembly being necessary. A convenient strategy consists on linking hydrophobic grafts to e.g., a highly water-soluble polysaccharide, inducing the formation of nanoparticles via hydrophobic interactions. This kind of amphiphilic polymers can be constructed by three routes: hydrophobic chains grafted to a hydrophilic backbone, hydrophilic chains grafted to a hydrophobic backbone (grafted polymers) or with alternating hydrophilic and hydrophobic segments (block polymers). Upon contact with an aqueous environment, amphiphilic polymers spontaneously form self-aggregated nanoparticles, via intra- or intermolecular associations between the hydrophobic moieties, primarily to minimize the interfacial free energy. The important feature, from the physicochemical point of view, is that the molecule is able to orient itself to expose the hydrophilic regions to the polar environment (normally the aqueous medium) and the hydrophobic segments aggregates in the internal core of the material. The concentration above which the polymeric chains aggregate is called the critical micelle concentration or the critical aggregate concentration. Several strategies for the preparation of nanoparticles have been used, including direct dissolution, solvent evaporation/film formation, dialysis, emulsion, and co-solvent evaporation. The commercialization of nanodevices obtained using these technologies is limited due to the employment of potentially toxic organic solvents and surfactants, often not acceptable, at least for parenteral administration. For example, polymer based nanoparticles may be prepared by the well-known solvent evaporation method, in which the droplets of a nanoemulsion are composed of a volatile organic solvent in which the polymer is solubilized [28]. Traces of solvent are very difficult to eliminate, even using very sophisticated and time consuming methods such as ultradialysis, ultracentrifugation or ultrafiltration. In general, the currently available nanotechnologies are not able to meet the severe requirements enacted by the public health agencies for medicines. Therefore, there is an urgent need to develop new concepts and ideas to overcome these technological issues by proposing preparation procedures avoiding the use of organic solvents and surfactants. In this view, direct dissolution in pure water is the most suitable strategy to obtain nanogels [29].

## 3. Drug Loading, Targeting and Release

### 3.1. Drug loading

Nanogels are widely used as carriers of therapeutic agents. A successful nanodelivery system should have a high drug-loading capacity, thereby reducing the required amount of carrier. Therapeutic agents can either be physically entrapped into the polymeric matrix or covalently bound to the polymer backbone. Polysaccharides, for instance, contain hydroxyl groups that allow direct reaction with drugs containing carboxylic acid function, producing ester linkages. Drugs lacking a carboxylic acid group require activation before the reaction takes place [30,31]. Physical drug entrapment is by far the more often used loading method for drug delivery applications. The best incorporation strategy for an efficient entrapment must be selected according to the physicochemical characteristics of the pair drug-carrier. Several methods have been used for drug loading, such as dialysis, nanoprecipitation, solvent displacement/evaporation, desolvation or direct dissolution. The encapsulation efficiency is different for each specific nanosystem; for example, the anti-cancer taxol is encapsulated with 100% and 20% efficiency on poly(lactide-co-glycolide) [32] and poly(ε-caprolactone) [33] nanodevices, respectively.

The incorporation of biomolecules without compromising its bioactivity constitutes a fundamental goal. Physical drug loading can be performed by incorporating the drug while producing the nanoparticles, or by incubating a concentrated drug solution with the already formed nano-carrier. Drug loading and entrapment efficiency depend on the solubility in the polymeric matrix, which is in turn related to the polymer composition, molecular weight, drug-polymer interactions, and the presence of functional groups (*i.e.,* ester or carboxyl) [34,35,36].

### 3.2. Targeting

**Figure 4:**
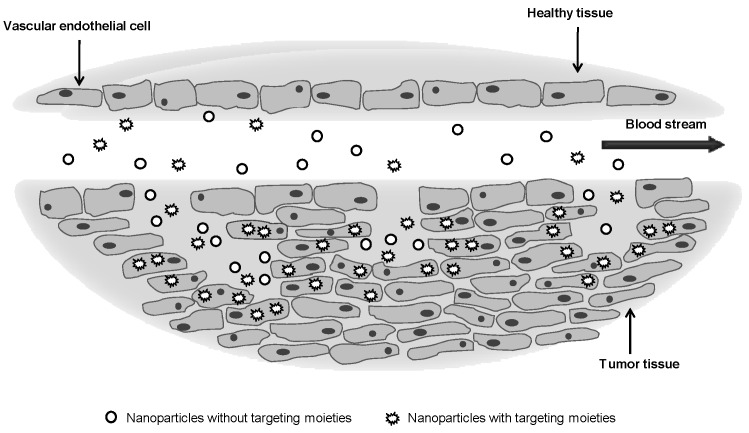
Targeting strategies for cancer therapy. Passive targeting can be achieved by enhanced permeation and retention, an effect involving leaky vascular structures. Active targeting mediated by targeting ligands specifically localizes drug carriers at desired cells or tissues. The decoration of the nanoparticles with ligands improves its internalization by endocytosis.

Drug delivery mediated by nanoparticles can be either an active or passive process. Passive delivery refers to the transport through leaky capillary fenestrations, into e.g., tumor interstitium and cells, by passive diffusion [37]. Selective accumulation of nanoparticles and drug then occurs by EPR effect. Active targeting involves the use of peripherally conjugated targeting moieties, for enhanced delivery to a specific site, based on molecular recognition. One such approach is to surface-coat nanoparticles with an antibody, which can interact with its specific antigenic target cell site. According to some authors, antibody targeting does not increase tumor localization, instead increasing internalization [38,39]. The targeting moieties may help cellular uptake. Thus, long circulation times will allow for effective transport of the nanoparticles to the tumor site through the EPR effect, the targeting molecule then enabling endocytosis of the nanoparticles (Figure 4). The internalization of the nanoparticle is important for effective delivery of bioactive agents, especially in gene delivery, gene silencing, and other biotherapeutics. Sophisticated strategies to control the intracellular trafficking of nanoparticles are being developed [40,41]. Such advanced drug carriers can be intelligently designed based on the inherent properties of the target site such as pH, presence of enzymes or specific tissue markers. After internalization by endocytosis, nanoparticles end up in endosomes and then lysosomes, where the pH values of about 5.5 (endosomes) and 4–5 (lysosomes) are found [42]. Thus, pH sensitive constructs may be used as a smart trigger system for drug release.

For optimal therapeutic effect, the drug concentration must be maintained at an effective dose at the target site for the appropriate time-frame, with minimal dose accumulation at off-target sites. For these purposes, careful design of multifunctional drug carriers with nano-dimensions has become a popular research subject.

Antibody fragments containing only the variable region of the antibody—that governs the recognition specificity—are now more commonly used for active targeting of therapeutics, avoiding the presence of the constant Fc effector region. This region may trigger the complement activation or undesirable interaction with other cells, potentially leading to premature phagocytosis of the drug delivery system [43]. In addition, the smaller size of antibody fragments may be an important factor in the development of an actively targeting nanoparticle. Antibodies, or antibody fragments, are conjugated either directly to the nanoparticle surface or through linker molecules such as PEG. The conjugation reaction is commonly carried out using carbodiimide-mediated chemistry, which creates stable amide bonds between carboxylic acid groups in the nano-carrier and primary amine groups, including lysines and the N-terminus amine, in the antibody or antibody fragment [43].

Cell-specific ligands have been popularly employed to confer tissue- or cell-specificity to drug delivery systems. Specific interaction between a targeting ligand and its cellular receptor generally enhances the cellular uptake of nano-carriers by a mechanism called receptor-mediated endocytosis. Therefore, increasing the specific cellular uptake significantly improve the therapeutic efficacy achieved with a much lower dose [44]. A variety of targeting ligands have been used in drug delivery systems, including galactose and lactose to target hepatocytes, mannose for dendritic cells, RGD peptide to target integrins on cell surfaces and lactoferrin for bronchial epithelial.

Different targets may be addressed for cancer therapy purposes. Targeting angiogenesis has become a major area of focus. The growth of solid tumors is dependent upon the ability to generate an adequate blood supply. Anti-angiogenesis approaches are effective in limiting tumor growth, the transformed endothelial cells of the neovasculature becoming a preferential target. Main angiogenic targets are the vascular endothelial growth factor receptors, α_v_β_3_ integrins, matrix metalloproteinase receptors, and vascular cell adhesion molecule-1. Cell proliferation markers are another important target for cancer therapeutics, as many of them are highly overexpressed by certain tumor cells. Actively targeting nanoparticles to cell proliferation receptors may be achieved using monoclonal antibodies. The selection of monoclonal antibodies for cancer targeting is based on four basic criteria: (1) the antigen of interest is overexpressed by tumor cells, (2) the antigen participates as a principle component in the progression of the disease, (3) the antigen, present on the tumor cell surface must be stable, and (4) the antigen is expressed by a large percentage of tumor cells and a large variety of tumors. The most established cell proliferation targets used by actively targeting nanoparticles include human endothelial, transferrin and folate receptors. Targeting highly expressed antigens is a promising area that can ensure the elimination of malignant tumors and metastatic cells that have not become large enough to induce angiogenesis.

### 3.3. Drug release

The nanoparticles made of biodegradable materials allow sustained drug release over periods of days or even weeks. Biodegradation should not only modulate the release of drugs for a desired period of time, but also enable the removal of the empty device. Drug release is affected by the particle size. Smaller particles have a larger surface-to-volume ratio, therefore most of the drug is at or near the particle surface, leading to faster drug release. In contrast, larger particles allow more drug to be encapsulated in the inner cores, providing a slower release. Thus, control of particle size provides a means of tuning the drug release rates. The release mechanism can be modulated also by the molecular weight and the copolymer composition used. It has been shown that the higher the polymer molecular weight, the slower the *in vitro* release of drugs [37,45]. The release mechanism is a complex function of three main processes, *i.e.*, drug diffusion, matrix swelling, and chemical reactivity of the drug/matrix. Recent studies focuses on biopolymers responsive to physiological changes such as pH, temperature and external stimuli such as light, that can trigger a control release of the therapeutic agent.

## 4. Applications

### 4.1. Small molecular weight drug delivery

The sustained delivery of small drugs from polymer formulations has broad application in the treatment of several diseases—namely and noteworthy, the case of cancer. Biomaterial-mediated delivery schemes offer a unique method to deliver this kind of drugs. This section summarizes the polymer-based delivery systems for small drugs described in the literature, namely studies *in vivo* or using cell lines (Table 1).

**Table 1:** Nano-carriers for small molecular weight drug delivery.

Polymer	Ligand	Target	Cell line	Drug binding	Stimuli	Ref
azo-dextran	aspirin	--	COS-1	simple mixing and irradiation with UV light	UV–vis light	[46]
carboxymethyl chitosan-linoleic acid	adryamycin (anti-cancer)	--	HeLa	direct dissolution	--	[47]
pullulan acetate	adriamycin (anti-cancer)	vitamin H (biotin)	HepG2	dialysis method	--	[48]
pullulan acetate/sulfonamide	adriamycin (anti-cancer)	--	MCF-7	dialysis method	pH	[49]
poly[(maleilated dextran)-*graft*-(*N*-isopropylacrylamide)]	camptothecin (anti-cancer)	--	L929	dialysis method	pH, temperature	[50]
poly(N-isopropylacrylamide)/chitosan	camptothecin (anti-cancer)	--	SW480	direct dissolution	pH	[51]
poly[2-(*N*,*N*-diethylamino)ethyl methacrylate]-*block*-PEG	cisplatin (anti-cancer)	--	SKOV-3 *In vivo*	solvent displacement method	pH	[52]
poly (lactide-co-glycolide)-PEG	curcumin (anti-cancer)	--	KBM-5, Jurkat, DU145, MDA-MB-231, HCT116,SEG-1 *In vivo*	nanoprecipitation	--	[53]
polylactide-*co*-glycolide–PEG–folate	docetaxel (anti-cancer)	folate	SKOV3	emulsification/solvent diffusion method	--	[54]
poly(D,L-lactic-*co*-glycolic acid)-*block*-PEG	docetaxel (anti-cancer)	PSMA aptamer	LNCaP	nanoprecipitation	--	[55]
glycol chitosan-5β-cholanic acid	docetaxel (anti-cancer)	--	A549 *In vivo*	dialysis method	--	[56]
poly(l-histidine)-*b*-PEG-folate (75 wt.%) and poly(L-lactide)-b-PEG-folate (25 wt.%)	doxorubicin (anti-cancer)	folate	MCF-7	. dialysis method	pH	[57]
chitosan-poly(acrylic acid)	doxorubicin (anti-cancer)	--	HepG2 *In vivo*	direct dissolution	--	[58]
poly(ε-caprolactone)-PEG-poly(ε-caprolactone)	honokiol (anti-inflammation)	--	A549	direct dissolution	--	[59]
ethylcellulose methylcellulose	nimesulide (nonsteroid anti-inflammation)	--	fresh human blood	desolvation method	--	[60]
poly(ethylene oxide)-modified poly(ε-caprolactone)	tamoxifen	--	MDA-MB-231 *In vivo*	solvent displacement	--	[61]
PEG-polycyanoacrylate	paclitaxel (anti-cancer)	transferrin	*In vivo*	solvent evaporation	--	[62]
poly (lactide-co-glycolide fumarate)/poly(lactide-co-ethylene oxide fumarate) poly (lactide-fumarate)/ poly(lactide-co-ethylene oxide fumarate)	paclitaxel (anti-cancer)	--	HCT116 *In vivo*	dialysis method	--	[63]
PEG750-block-poly(ε-caprolactone-co-trimethylenecarbonate)	paclitaxel (anti-cancer)	--	HeLa *In vivo*	solvent evaporation	--	[64]
poly (lactide-co-glycolide)/ poly(ε-caprolactone)-PEG	paclitaxel (anti-cancer)	--	HeLa	simple emulsion or nanoprecipitation method	--	[65]
linoleic acid/poly(β-malic acid) Chitosan	paclitaxel (anti-cancer)	--	*In vivo*	sonication and dialysis method	--	[66]
poly(β-amino ester)-graft-PEG	paclitaxel camptothecin (anti-cancer)	--	SKOV-3	solvent displacement or dialysis method	pH	[42]
glycol chitosan-5β-cholanic acid	protophorphyrin IX (photosensitizer, photodynamic therapy)	--	SCC7 *In vivo* *Ex vivo*	dialysis method	--	[67]
poly(β-benzyl-l-aspartate)-*block*-poly(vinylpyrrolidone)	prednisone (anti-inflammation)	--	SW-1990	dialysis method	pH	[68]
poly (10-undecenoic acid-b-N-isopropylacrylamide)	prednisone (anti-inflammation)	--	ECV304	dialysis method	pH, temperature	[69]
cellulose-*g*- poly(l-lactide)	prednisone (anti-inflammation)	--	3T3	dialysis method	--	[70]
galactosylated polycaprolactone-g-dextran	prednisone (anti-inflammation)	galactose	HepG2, 3T3 *In vivo*	dialysis method	--	[71]
poly(ethylene oxide)-modified poly(ε-caprolactone)	saquinavir (HIV-protease inhibitor)	--	THP-1	solvent displacement	--	[72]
water soluble chitosan	thymol (anti-microbial)	--	*Staphylococcus aureus* *Bacillus subtilis* *Escherichia coli* *Aspergillus niger*	sonication method	--	[73]

Cell line abbreviations: human cervix epithelial carcinoma cells (HeLa), human hepatocellular carcinoma cells (HepG2), breast carcinoma cells (MCF-7), mouse fibroblast cells (L929), human colon carcinoma cells (SW480), human epithelial ovarian cancer cells (SKOV-3), human chronicmyeloid leukemia (KBM-5), human T cell leukemia (Jurkat), prostate carcinoma (DU145), breast adenocarcinoma (MDA-MB-231), human colon adenocarcinoma (HCT116), human esophageal adenocarcinoma (SEG-1), prostate epithelial cells (LNCaP), lung cancer cells-bearing mice (A549), human colon carcinoma cells (HCT116), squamous cell carcinoma cells (SCC7), pancreatic cancer cells (SW-1990), human vein endothelial cells (ECV304), mouse fibroblasts (3T3), human monocyte/macrophage, myelomonocytic cells (THP-1)

Several polymers have been exploited for the formulation of nanoparticulate carrier systems. PLA, PGA and PLGA have been extensively employed because of their biocompatibility, biodegradability and versatile degradation kinetics. For instance, haloperidol-loaded PLGA/PLA particles were produced by sonication or homogenization. The three most relevant properties regarding the release behavior were identified: polymer hydrophobicity, surface coating and particle size. The hydrophobicity of the polymer contributes to a reduction of the initial burst, extending the period of release. For example, the percentage of drug released after 1 and 35 days is respectively 46 and 70% for 220 nm PLA particles, as compared to 70 and 90% for PLGA particles with the same size. Coating the particle surface with chitosan considerably reduces the initial burst. For example, the initial burst registered at 1 day, using 220 nm PLGA particles, is reduced from 70 to 36% by surface coating with chitosan. Increasing the size of the particles also reduces both the initial burst and the rate of release. For example, increasing the size from 220 to 450 nm reduces the initial burst from 48 to 28%, a steady release of drug being observed over a 10 day time period in the later case, as compared to four days for the smaller particles [74].

PEG has been extensively used to modulate the biodegradation, release profile, bloodstream clearance and biodistribution. For instance, nanoparticles engineered by blending PLA/PLGA homopolymers and PEG have been used to encapsulate betamethasone disodium 21-phosphate (BP). The drug release profile and the vascular circulation time-frame could be controlled by varying the composition/molecular weight of the polymer. The rate of *in vitro* BP release correlated inversely with the nanoparticles size and increased using PLGA instead of PLA homopolymers. Furthermore, higher PEG content reduces the uptake of nanoparticles by dendritic cells and accordingly improves the blood circulation time. Analysis of the BP organ biodistribution revealed a lower liver concentration when using blended nanoparticles instead of PLA nanoparticles [75]. The *in vitro* degradation of the PLGA–mPEG nanoparticles increases with the proportion of mPEG in the copolymer chains. As expected, the hydrophilicity of PLGA–PEG copolymers, as evaluated by water uptake or contact angle measurements, was found to increase with an increase in PEG content. The higher degradation rate of the nanoparticles with high mPEG content may be attributed to the improved hydrophilicity, which apparently overrides the decreased content of cleavable ester bonds [76].

### 4.2. Protein, peptide and oligosaccharide delivery

The oral route is the most convenient and comfortable mean for drug administration. However, the poor bioavailability, mainly due to the low mucosal permeability and lack of stability in the gastrointestinal (GI) environment, resulting in compound degradation prior to absorption, hinders a generalized use for proteins and peptides administration. One possible way to improve the GI uptake of proteins and peptides is the encapsulation in micro/nanoparticles that, while protecting from degradation in the GI tract, facilitate the transportation into the systemic circulation. Polymeric nanoparticles meet these attributes. [77,78].

The rather large number of therapeutic proteins currently reaching the market or under clinical evaluation draws the urgent need for new delivery systems, using different routes of administration, which allow for improved stability—a main concern regarding therapeutic proteins—as well as controlled and targeted release. Cytokines emerge as a powerful tool for immunotherapy and vaccination purposes. Simultaneously, growth factors offer new opportunities for wound and tissue regeneration. Currently, the more effective approach to guarantee appropriate stability is protein pegylation. Other tools with more versatile properties are required. Colloidal polymeric carriers have arisen as a promising alternative for improving the transport of macromolecules:

- proteins such as insulin [79,80,81,82,83];
- cytokines [84,85] and growth factors [86];
- peptides, e.g. RGD [87], cyclosporin A [88,89], elcatonin [90,91], vasoactive intestinal peptide [92], anti-cancer glycoprotein lectin A-chain [93] and a caspase inhibitor peptide [94]
- oligossacharides, such as heparin [95,96,97].

These recent reports on the development of new protein delivery systems are discussed below.

The cationic nature of chitosan makes it an interesting polymer for the association with and delivery of labile negatively charged macromolecules. Several studies addressed the nasal delivery of insulin with chitosan nanoparticles [98,99]. The potential of protonated chitosan has been enforced by the recognition of its ability to trigger the opening of tight junctions, thereby facilitating the transport of macromolecules through the epithelium. Indeed, it has been demonstrated that nanoparticles can enhance the oral bioavailability of encapsulated therapeutic proteins. For this purpose, a nanoparticulate system composed of trimethyl chitosan (TMC) and cysteine (Cys), attempting to combine the mucoadhesion and permeation enhancing effects, was tested. When reaching the small intestine, the positively charged nanoparticles—which interact electrostatically with the mucous layer—induce transient loosening of the tight junctions. The free thiol groups on TMC-Cys allow the formation of disulfide bonds with the cysteine-rich mucin. Therefore, a closer and prolonged action on the tight junctions favors both the paracellular transport of the insulin and nanoparticle internalization by enterocytes. Oral administration of insulin-loaded TMC-Cys nanoparticles led to notable hypoglycemic effects, which lasted until eight hours post-administration, with a maximum blood glucose depression of 35% [100].

In cytokine immunotherapy, a suitable delivery system that ensures slow-release of cytokines is required, the short *in vivo* half-life of these molecules ruins its therapeutic efficacy, while causing severe systemic toxic effects. Recombinant murine IL-12 (rmIL-12) was successfully incorporated into cholesterol-bearing pullulan (CHP). The subcutaneous injection of the CHP/rmIL-12 complex led to a prolonged elevation of IL-12 concentration in the serum. Repetitive administrations of the complex induced drastic growth retardation of reestablished subcutaneous fibrosarcoma, without causing toxicity [84].

Bone morphogenetic proteins (BMPs) are cytokines with a strong ability to promote new bone formation. Elastin-like nanoparticles, created by thermoresponsive self-assembly, were developed for the combined release of bone morphogenetic protein-2 (BMP-2) and bone morphogenetic protein-14 (BMP-14). These BMPs could be encapsulated efficiently into the elastin-like particles and delivered in a sustained way for 14 days. The activity of the growth factors was retained and increased bioactivity on C2C12 cells was observed following the combined release of BMP-2 and BMP-14 [86].

The development of peptides and proteins acting on the central nervous system is drastically hindered by the blood-brain barrier (BBB). The surface engineering of nanoparticles with lectins opened a novel pathway for the delivery of drug-loaded biodegradable nanoparticles into the brain, following intranasal administration. The neuroprotective Vasoactive Intestinal Peptide (VIP) was efficiently incorporated into PEG-PLA nanoparticles surface-modified with wheat germ agglutinin. This formulation allows a more effective delivery, as compared with the intranasal application of the soluble peptide. This is partially attributed to the higher affinity of the wheat germ agglutinin-conjugated nanoparticles to the olfactory mucosa, rather than to the respiratory one [92]. The peptide Z-DEVD-FMK, a caspase inhibitor, reduces vulnerability of the neuronal cells. The clinical application is hindered by its inability to cross the BBB and diffuse into the brain tissue. Thus, chitosan-PEG nanospheres bearing the OX26 monoclonal antibody (affinity for the transferrin receptor) have been designed, which trigger the receptor-mediated transport across the BBB. An important amount of nanoparticles were located in the brain, outside of the intravascular compartment. Hence, OX26 functionalized chitosan-PEG nanoparticles are promising carriers for the transport of the anti-caspase peptide into the brain [94].

The peptide Arg–Gly–Asp (RGD) draws much attention for tumor therapy applications, because it specifically binds α_v_β_3_ integrins, expressed by angiogenic endothelial cells. Attempting to overcome the short half-lives of several systemic anti-angiogenic peptides, a carrier for the RGD peptide was produced, using self-assembled nanoparticles of hydrophobically modified glycol chitosan (HGC). A high RGD loading efficiency was achieved (over 85%), and the system showed prolonged and sustained release for about 1 week. The RGD loaded-HGC nanoparticles displayed anti-angiogenic activity, markedly suppressing bFGF (inducer of angiogenesis) as well as preventing microvessel formation. Due to the sustained RGD peptide delivery, RGD-HGC nanoparticles significantly decreased tumor growth and microvessel density, improving the effect obtained injecting the free RGD peptide, either intravenously or intratumorally [101].

Limitations in the management of extraocular diseases include the inability to provide long-term extraocular drug delivery without compromising intraocular structures. Since the cornea has negative charge, mucoadhesive polymers can interact with the extraocular structures, increasing the concentration and residence time of immunosuppressive peptide cyclosporin A (CyA). The hydrophobic peptide, CyA, was associated to chitosan nanoparticles. *In vivo* experiments showed that, following topical instillation of CyA-loaded chitosan nanoparticles to rabbits, it was possible to achieve therapeutic concentrations in external ocular tissues (*i.e.*, cornea and conjunctiva) during at least 48 hours, while maintaining negligible or undetectable CyA levels in inner ocular structures (*i.e.*, iris/ciliary body and aqueous humor), blood and plasma [88].

Pulmonary drug delivery for both local and systemic action has many advantages over other delivery routes. The lungs offer a large surface area, a rather thin absorption barrier, low enzymatic metabolic activity and slow mucociliary clearance. Therefore, PLGA nanospheres, surface-modified with chitosan, were developed and used for pulmonary delivery of elcatonin, a calcitocin derivative (formulated for the management of several bone-related diseases). The coated nanospheres were eliminated from the lungs at slower rate. Loaded elcatonin reduced blood calcium levels to 80%, exhibiting a prolonged pharmacological action, for over 24 hours. The results showed that the nanospheres adhered to the bronchial mucus and lung tissue, allowing the sustained drug release at the adherence site. Additionally, flexible chitosan molecules on surface of the nanospheres enhanced the drug absorption, maybe by opening the intercellular tight junctions [90].

The macromolecular drug heparin, a sulfated natural glycosaminoglycan, is vulgarly used as an injectable anticoagulant [97]. Recently, heparin has been loaded into nanoparticles of chitosan and hyaluronic acid, developed for anti-asthmatic therapy applications. The nanosystems were stable in phosphate buffered saline pH 7.4 for at least 24 hours, and released 10.8% of unfractionated heparin within 12 hours of incubation. Fluorescent heparin-loaded nanoparticles were effectively internalized by rat mast cells. *Ex vivo* experiments, conducted to evaluate the capacity of heparin to prevent histamine release in rat mast cells, indicated that the free or encapsulated drug exhibited the same dose–response behavior [95]. Intracellular delivery of heparin by chitosan-g-PEG/heparin polyelectrolyte complexes has also been used to trigger caspase activation and consequently promote apoptotic death of cancer cells [96].

### 4.3. Vaccine delivery

The recent developments in nanotechnology increased considerably the interest in nanoparticulate systems as a platform for the delivery of antigens. The key challenges are the induction of a potent and broad (both humoral and cellular) antigen-specific immune response, capable of protecting from infection (sterilizing vaccination) and/or disease (therapeutic vaccination) and finally, to develop effective immunity after a single injection of the vaccine. Particulate delivery systems mimic pathogens that are commonly recognized, phagocytosed, and processed by professional antigen-presenting cells (APCs). APCs, such as dendritic cells and macrophages, represent the sentinels of the immune system and orchestrate antigen-specific T cell-mediated immune responses. Activated APCs migrate to regional lymph nodes where they present the antigen to T cells, thereby triggering cellular and humoral immunity. Most organisms are detected and destroyed within hours by defense mechanisms, which are not antigen-specific and do not require any prolonged period of induction. These are the mechanisms of innate immunity. Only when the infectious agent is capable of breaking this early line of defense, an adaptive immune response will develop. This includes generation of antigen-specific effector cells that specifically target the pathogen, secretion of antibodies (B cells), direct cytotoxic activity (T cells), or secretion of immunological mediators and effector molecules such as cytokines and chemokines. Although most of these effector cells will die within 10–14 days after infection, some cells will survive, as highly reactive plasma cells (B cells) or memory cells (B and T cells), and prevent subsequent infection by the same microorganism. Along with long-lasting antibodies against a specific pathogen, the induction of memory cells is the final goal of preventive vaccination.

Immunity against an infectious agent by vaccination can be achieved in various ways, normally including several components. Firstly, the antigen itself can be a synthetically produced peptide representing an epitope of a pathogen protein. It can also be the full-length protein carrying several epitopes that are recognizable by B and T cells. Such full-length proteins can be secreted from the pathogen or produced synthetically or recombinantly. Vaccine development has also focused on experimental vaccines where the gene encoding a particular protein is fused into a DNA or RNA plasmid. Secondly, the formulation or delivery system varies considerably from one vaccine to another. DNA and some protein vaccines can be administered in solution without adjuvant enhancement of the immune response. Today, still many of the most potent vaccines are given as a live attenuated or killed form of the particulate microorganism. A unique property, especially of live vaccines, is that they often induce strong T cell responses. This property is very much missed by protein or peptide vaccines administered with those adjuvants in general use today, e.g., aluminum salts. Thus, there is an urgent need for the development of potent and safe antigen-delivery systems. The development of nanoparticulate vaccines is also motivated by safety concerns, e.g., to avoid the risk of infection induced by live attenuated vaccines and to suppress the excessive inflammation that is frequently caused by the use of Freund’s adjuvant or aluminum salts adjuvant.

Cancer vaccines are a promising approach for anti-cancer therapy, as fewer side effects are induced than with other therapies and, more importantly, there is an opportunity for developing long-term immunity [102]. In contrast to general medicines, which are directly targeted to specific molecules, cancer vaccines initiate a cascade of antigen specific immune responses against antigen-expressing tumor cells. Activating the immune system to trigger a specific response sufficient for the eradication of tumor cells, however, is a major challenge in the development of cancer immunotherapy.

A possible key for the successful development of new generation of vaccines may lie in the use of targeted delivery systems (Table 2). Nanoparticulate systems are particularly well suited for the delivery of antigens specifically to DCs, inducing the subsequent activation of T cell immunity, given its ability to permeate the lymphatic draining system and reach DCs in the lymph nodes. However, antigen delivery to—and activation of—DCs is a complex problem, involving antigen transport to DC-rich areas, DC binding and eventually antigen uptake, and antigen processing and presentation. Before an antigen can be processed and presented by DCs, the biomaterial vehicle itself must be internalized by DCs. Immature DCs internalize exogenous solutes, particles, and necrotic or apoptotic cells through macropinocytosis, receptor-mediated endocytosis and phagocytosis [103]. Several studies confirmed that DCs can internalize polymeric nanoparticles [104], the internalization mechanism being partially controlled by altering the properties of the biomaterial vehicle, namely the size [105]. Macropinocytosis is used to internalize extracellular fluid and smaller solutes such as macromolecules [103] and particularly small nanoparticles (< 50 nm), whereas phagocytosis occurs when larger nanoparticles (> 500 nm) are taken up [106,107]. DCs also use surface receptors to endocytose ligands with a terminal sugar such as mannose [108]. Thus, both the physicochemical and biochemical character of biomaterial vehicles can be adjusted to tailor DC uptake.

Following internalization, the biomaterial vehicle must then release the antigen intracellularly, in a manner that will enable processing by MHC class I, class II, or both (cross-presentation) pathways. The delivery of exogenous antigen inducing cellular immunity through the MHC class I pathway can be a challenging problem, as internalized particles are initially within endosomes but are trafficked rapidly to lysosomes, where they are degraded enzymatically, preventing the antigen from being processed and presented. To avoid lysosomal trafficking, smart polymers have been designed. These polymers are broken down inside the endosomes, due to the presence of acid-degradable acetal bonds, triggering the disruption of the endosomes in a pH-dependent fashion. The process occurs as follows: at pH 7.4 the polymers are PEGylated (“masked”); however, after endocytosis the acid labile linker is hydrolyzed and the polymer backbone becomes de-PEGylated (“umasked”) and membrane-disruptive, causing endosomal disruption. The PEGs may be conjugated to the backbone via both acid-degradable linkages and disulfide bonds. These polymers release oligonucleotides and peptides into the cytoplasm as the endosome is acidified, avoiding the lysosomal fusion; releasing antigen into the cytoplasmic compartment enables processing by MHC class I instead of the MHC class II pathway [109]. This strategy exemplifies how smart biomaterials may be engineered as to overcome biological barriers and control the intracellular biodistribution.

Targeting lymph-node DCs, rather than peripheral ones in the skin for example, offers many theoretical advantages [110]. The avoidance of premature antigen presentation, because DCs in lymph nodes are already at the site of antigen presentation, is one potential benefit. For successfully targeting lymph-node-resident DCs, it is crucial to engineer biomaterial vehicles that can be readily taken up into lymphatic vessels after subcutaneous or intradermal injection, being then retained in draining lymph nodes. It has been well-established that particle size is among the most crucial factors for lymphatic uptake from the interstitial space [111].

As the mucosal route of administration (e.g., intranasal, oral) is considered a simple, safe, efficacious, non-invasive and less expensive method to deliver antigens, novel strategies for the achievement of safe and effective immunization strategies are under investigation regarding the routes of vaccination. Mucosal immunization is an attractive alternative to parenteral vaccination; using the appropriate delivery system it is possible to stimulate both mucosal and systemic immune responses [112]. Mucosal vaccination offers also several benefits over parenteral route, including ease of administration, reduced side effects, possibility of self-administration and, especially in developing countries, reduced risk of the unwanted spread of infectious agents via contaminated syringes.

**Table 2:** Polymeric nanoparticles for vaccine delivery.

Polymer	Antigen	Remarks	route	Ref
poly-l-lysine coated polystyrene particles	sOVA-C1 plasmid	Particles of different sizes may target different APCs.	intradermal	[113]
poly-(ε-caprolactone) -poly(lactide-co-glycolide)	diphtheria toxoid	Correlations between polymer characteristic (e.g., hydrophobicity ) and route of administration, indicate that such characteristics can have interesting implications in immune responses.	intranasal intramuscular	[114]
methoxyPEG–poly(lactide-co-glycolide)	recombinant hepatitis B surface antigen (HBsAg)	Delivery of HBsAg encapsulated within a nanoparticle is a superior way for generating faster immune responses, as compared to the non-encapsulated counterpart.	intraperitoneal	[115]
poly lactic acid-PEG	recombinant hepatitis B surface antigen (HBsAg)	Different compositions of PLA and PEG polymers were synthesized to stabilize the antigen. A comparison of their efficacy in the generation of an effective immune responses is shown.	nasal	[5]
poly(γ-glutamic acid)-graft-L-phenylalanine	japanese encephalitis (JE) vaccine	A single dose of JE vaccine with nanoparticles enhanced the neutralizing antibody titer.	intraperitoneal	[116]
poly(γ-glutamic acid)-graft-l-phenylalanine	influenza hemagglutinin (HÁ) vaccine	Subcutaneous immunization with a mixture of HA vaccine and nanoparticles induced higher mononuclear cell proliferation and production of IFN-γ, IL-4, and IL-6 upon HA restimulation.	subcutaneous	[117]
poly (d,l-lactide-co-glycolide)–polyethyleneimine	DNA encoding Mycobacterium tuberculosis latency antigen Rv1733c	The polyplexes were able to mature human dendritic cells and stimulated the secretion of cytokines, comparable to levels observed after lipopolysaccharide stimulation.	intramuscularly endotracheal	[118]
hydrophobically modified poly(γ-glutamic acid)	gp120 (human immunodeficiency virus -1)	The protein-encapsulated nanoparticles induced cytotoxic T lymphocyte. Efficient uptake by immature dendritic cells (DC) and induction of DC maturation was observed.	intranasal	[25]
chitosan	DNA vaccine encoding mite dust allergen Der p 2 (Der p 2-pDNA)	Chitosan-DNA nanoparticles can generate a higher level expression of gene *in vivo*, therefore can preferentially activate specific Th1 immune responses thus preventing subsequent sensitization of Th2 cell-regulated specific IgE responses.	oral	[119]
chitosan	plasmid DNA encoding surface protein of Hepatitis B virus (pRc/CMV-HBs(S))	Administration of nanoparticles resulted in serum anti-HBsAg titer and induced sIgA titre in mucosal secretions. Chitosan nanoparticles were able to induce humoral mucosal and cellular immune responses.	nasal	[120]
chitosan	DNA plasmids expressing different *M. tuberculosis* epitopes	Chitosan nanoparticles protect DNA from degradation by nucleases, induce dendritic cells maturation and increased IFN-γ secretion from T cells.	pulmonary	[121]
chitosan	pcDNA3-VP1, encoding VP1, major structural protein of coxsackievirus (CVB3)	Nasal administrated chitosan–DNA produced higher levels of serum IgG and mucosal secretory IgA. Strong cytotoxic T lymphocyte activities helped to effectively eliminate CVB3 viruses.	intranasal	[122]
low molecular weight chitosan (LMWC)	plasmid DNA encoding human cholesteryl ester transfer protein C-terminal fragment (CEPT)	LMWC had lower binding affinity to DNA, but mediated higher transfection efficiency. Polyplexes could elicit significant systemic immune responses, modulate the plasma lipoprotein profile and attenuate the progression of atherosclerosis.	intranasal	[123]
mono-N-carboxymethyl chitosan (MCC) N-trimethyl chitosan (TMC)	tetanus toxoid	Surface charge and particle size exert an important influence in the production of an enhanced immune response.	intranasal	[112]

### 4.4. Gene delivery

Nucleic acid-based biopharmaceuticals, such as pDNA, oligonucleotides (ODNs) and short interfering RNA (siRNA), are potential pioneering materials to cope with various incurable diseases. Cationic polymers condense DNA into nanosized polymer/DNA complexes (polyplexes), by a self-assembling process, consisting on the electrostatic interaction of the positively charged polymer with the negatively charged DNA. Polyplexes may interact with the negatively-charged cellular membrane, being internalized via endocytosis. In the intracellular environment, the polyplexes are normally located in endosomes that become acidified and finally fuse with lysosomes. In this case, DNA is prone to degradation by lysosomal enzymes. In order to transfer their DNA cargo successfully to the nucleus, polyplexes must escape from the endosome. After endosomal escape, polyplexes are located in the cytoplasm, ready to unpack DNA and deliver it to a suitable site near the nucleus or in the nucleus. Finally, after the DNA translocation into the nucleus, gene expression must occur. The low efficiency of polymer-mediated gene delivery may be due to the lack of mechanisms to overcome the physiological barriers. Cationic polymers need to have multiple functions to overcome these barriers, such as good DNA binding ability to condense DNA into polyplexes, high buffer capacity to induce endosomal escape and efficient intracellular vector unpacking to release DNA [124]. Therefore, understanding of the correlations between polymeric functionalities and gene delivery properties is important for the rational design of efficient cationic polymeric vectors.

RNA may be advantageously used instead of DNA for gene delivery purposes. First, the delivery target is the cytosol, not the nucleus. Cytosol delivery is by far easier and more efficient than nucleus delivery. Quite recently, siRNA has emerged as a more powerful therapeutic genetic agent [125]. High sequence specificity and relatively small dose requirement of siRNA make it even more attractive. The most challenging hurdles are serum instability during circulation in the bloodstream [43], poor cellular uptake, and limitation in targeted delivery to specific tissues or cells. To address such problems, natural or synthetic polymeric delivery systems have been used. The natural polymers investigated for gene therapy include chitosan, collagen, gelatin and their modified derivatives [126] (Table 3). Among the synthetic polymers, poly(l-lysine) or polyethylenimine and their analogs have been widely used [127] (Table 4). Other synthetic alternative nano-carriers have been synthesized, engineered with linkages envisaging physiological degradation (Table 5). Specific targeting moieties may be conjugated to confer tissue specificity. Chitosan has emerged as an alternative non viral gene delivery system. The transfection efficiency of chitosan/DNA complexes is dependent on several factors: chitosan degree of acetylation and molecular weight [128,129,130], amine/phosphate ratio of chitosan/DNA complexes [131,132], serum concentration, pH [128] and cell type [133,134]. Chitosan derivatives (glycol, o-carboxymethyl, trimethylated, thiolated and 6-N,N,N-trimethyltriazole chitosan) including hydrophobic modifications (deoxycholic acid, 5β-cholanic acid, N-acylated chitosan) have been used to overcome the limited chitosan solubility and improve transfection efficiency. Concerning biodegradation, chitosan is degraded into oligomers by lysozyme, and then further degraded by N-acetyl-glucosaminidase, in animal cells. Both of these enzymes are present in the endosomal/lysosomal vesicles, thus the degradation and release of pDNA will start immediately after endocytosis of the chitosan polyplexes. Additionally, lysozyme is present at inflammation sites, which allow specific release. The chitosan cytotoxicity is lower than that of polyethylenimine, supporting the suggestion that chitosan may be a nontoxic alternative to polyethylenimine [135].

Polyethylenimine (PEI), often considered the gold standard of gene transfection, is one of the most prominent examples of cationic polymers capable of gene transfection. The transfection efficiency of PEI has been related to the buffering effect exerted by the amines, with different pKa values, over a wide range of pH. This buffering ability gives PEI an opportunity to escape the endosome (proton sponge effect) [136]. PEI, as poly-L-lysine (PLL), often show a relatively high cytotoxicity and, depending on the ionic strength, a tendency to aggregate and precipitate [137]. The conjugation with PEG prevents the inter-particular aggregation of the complexes, increasing their stability [138,139].

The non-degradability of non-viral carriers may represent a major limitation, since it implies they are not removed by physiological clearance systems and, therefore, can possibly accumulate within cells or tissues, eliciting further cytotoxicity. The backbone linkages of most polymeric gene carriers consist of a –C–C– bond or amide bond, which are not degraded in physiological solutions. Additionally, the biodegradation of the polymer may provide an extra tool to release the plasmid DNA into the cytosol. In an effort to develop alternative non-toxic and effective nano-carriers modified polymers, with functional groups labile under physiological conditions (ester, phosphorus or disulfide bonds), were produced. The use of the disulfide bond as bioreducible linker has received much attention in recent years [140,141]. The disulfide bond can be cleaved intracellularly by reducing enzymes such as glutathione reductase and sulfhydryl components like glutathione. Since the concentration of these reducing species is much higher in the cytoplasm than in plasma (intracellular *versus* extracellular glutathione concentration 0.5–10 mM *versus* 2–20 μM) [142], the disulfide bond is relatively stable in the extracellular environment, but rapidly degradable inside the cells, due to the higher amounts of thiols. To achieve high efficiency of polymer-mediated gene delivery, endosomal escape is required. This mechanism can be improved with the addition of chloroquine or membrane-active peptide [143]. An alternative method to promote endosomal lysis consists in engineering the carrier cationic polymers with histidine or imidazole groups. The transfection enhanced activity is assigned to the imidazole heterocycle, which displays a pKa around 6, thus possessing a buffering capacity at the endolysosomal pH [144].

Several examples of polymeric nanogels developed for gene therapy are given below.

Anti-angiogenic therapy has become an important route for cancer treatment. Among factors that regulate angiogenesis, the vascular endothelial growth factor (VEGF) appears to be the most critical regulator of tumor-induced angiogenesis, which is essential for the survival of rapidly proliferating cancer cells and sustained growth of tumor. For this purpose, a PEG-conjugated VEGF-siRNA was complexed with polyethylenimine. Intravenous as well as intratumoral administration of these polyplexes significantly inhibited VEGF expression at the tumor tissue, suppressing tumor growth in an animal tumor model, without showing any detectable inflammatory responses in mice [145].

**Table 3:** Natural-based polymers for gene delivery.

Polymer	Therapeutic agent	Ligand	Remarks	Ref
chitosan	sense or antisense oligodeoxynucleotides (ODNs) against malarial topoisomerase II gene	--	Antisense-nanoparticles demonstrate a significant higher inhibition of human malaria parasite, as comparison with sense-nanoparticles and free ODNs. More easily dissociated complexes mediate a faster onset of action.	[146]
folate-N-trimethyl chitosan	pDNA	folate	Folate conjugation increased intracellular uptake , transfection efficiency and induce endosomal escape.	[147]
folic acid-chitosan	pDNA (pVR1412)	folate	Nanoparticle with positive zeta potentials interact with the cell membrane allowing their endocytosis.	[148]
galactosylated 6-amino-6-deoxychitosan	pDNA (pCMV-Luc)	galactose	The increase of transfection efficiency of Gal-6ACT was therefore likely due to improvements in intracellular trafficking and not due to the increase of cellular uptake.,	[149]
chitosan/hyaluronic acid	pDNA(pEGFP-C1, pβ-gal)	hyaluronan	Polyplexes were able to provide high transfection without affecting cell viability, entering the corneal epithelial cells by CD44 receptor–mediated endocytic uptake.	[150]
mannosylated chitosan	pDNA (pGL3-Luc)	mannose	Cellular uptake mediated by mannose recognition. Reduced toxicity and high transfection efficiency.	[151]
chitosan –IL-1Ra folate- IL-1Ra- Chitosan	IL-1Ra plasmid DNA	folate	Folate-chitosan-DNA nanoparticles containing the IL-1 Ra gene prevent bone damage and inflammation in rat adjuvant-induced arthritis model that overexpress folate receptors.	[152]
PEG-Chitosan	pDNA (pRE-luciferase; pREP4;pCMV-luciferase)	transferrin KNOB protein	The transfection efficiency was much impressive with KNOB (130-fold improvement), in HeLa cells. Chitosan exhibited limited buffering capacity. The clearance of the PEGylated nanoparticles was slightly slower than that of the unmodified nanoparticles.	[153]
chitosan	plasmid pGL3-Luc	--	Polyplexes are endocytosed and possibly released from endosomes due to swelling of both lysosomes and polyplexes, causing the endosome rupture.	[154]
chitosan	pDNA (pAAV-tetO-CMV-mEpo and pCMVβ)	--	Oral gene therapy was efficient in delivering genes to enterocytes.	[155]
thiolated chitosan	pDNA (pEGFP-N2)	--	Improved gene delivery *in vitro* as well as *in vivo*. The extended pDNA release and subsequent gene expression were achieved by oxidation of introduced thiol groups to crosslink the thiolated chitosan.	[156]
quaternized (trimethylated) chitosan oligomer	pDNA (pEGFcp1-GFP)	--	Transfection efficiency decreases increasing the degree of quaternization. The polymer effectively transfers the GFP gene into cells both *in vitro* and *in vivo*.	[157]
6-*N,N,N*-trimethyltriazole chitosan	pDNA (EGFP-N1	--	The presence of the trimethyltriazole group led to significantly increased cellular uptake, which resulted in higher transfection efficiency in HEK 293 and MDA-MB-468 cells.	[158]
methoxy PEG–PEI–chitosan	pDNA (pVRfat-1)	--	The mPEG increased the slow-releasing ability and water solubility, while PEI improved the transfection efficiency.	[159]
chitosan/ poly(γ-glutamic acid)	pDNAs (pEGFP-N2, pGL4.13 and pEGFP-N2)	--	The incorporation of γ-PGA in the chitosan nanoparticles facilitates the dissociation of chitosan and DNA, increasing transfection efficiency. Trypsin-cleavable proteins in cellular membrane affect internalization of polyplexes.	[160]
methylated collagen	pDNA (pRELuc)	--	Methylated collagen improved DNA binding ability and the stability of the complexes at physiological conditions, as compared with unmodified native collagen.	[161]
cationized gelatin	plasmid DNA of transforming growth factor-βR (TGF-*β*R) siRNA expression vector	--	The injection of polyplexes significantly decreased the level of TGF-*β*R and α-smooth muscle actin over-expression, the collagen content of mice kidney, and the fibrotic area of renal cortex, in contrast to free plasmid DNA injection.	[162]
PEG–modified thiolated gelatin	pDNA (EGFP-N1)	--	Nanoparticles released encapsulated plasmid DNA in response to varying concentrations of glutathione.	[163]

Multidrug resistance remains a major barrier to the success of anti-cancer chemotherapy [164]. Overexpression of drug efflux transporters, such as P-glycoprotein, enables cancer cells to develop resistance to multiple anti-cancer drugs. A novel approach to overcome drug resistance consist in using the siRNA-mediated silencing the expression of the efflux transporter. Because P-glycoprotein plays an important role in the physiological regulation of endogenous and xenobiotic compounds, it is important to deliver the P-glycoprotein targeted siRNA (associated with anti-cancer drugs) specifically to tumor cells. Recent studies have shown that nanoparticles formulated with poly(d,l-lactide-co-glycolide) and polyethyleneimine result in sustained siRNA delivery and efficient gene silencing Nanoparticles were surface functionalized with biotin for active tumor targeting. *In vivo* studies, in a mouse model of drug-resistant tumor, demonstrated significantly greater tumor growth inhibition following treatment with biotin-functionalized nanoparticles encapsulating both paclitaxel and P-glycoprotein targeted siRNA. Remarkably, this effect was obtained using a paclitaxel dose ineffective in the absence of gene silencing [165]. Recently, dual nanogels, for co-delivery of drug and gene, were synthesized and evaluated as carriers [166,167]. Inhibition of ret/PTC1 oncogene, in the papillary thyroid carcinoma, has been achieved after administration of siRNA using chitosan-coated biodegradable poly(isobutylcyanoacrylate) nanoparticles. The nanoparticles protect the ret/PTC1 siRNA from *in vivo* degradation, leading to significant tumor growth inhibition after intratumoral administration, correlated to reduced ret/ PTC1 levels [168].

A novel approach for the control of inflammation in rheumatoid arthritis was reported. The strategy consisted in using chitosan/siRNA nanoparticle to silence the TNF-α expression in peritoneal macrophages. The nanoparticles, containing an unmodifed anti-TNF-α DsiRNA, mediated TNF-α knockdown (~66%) in primary peritoneal macrophages,* in vitro*. Histological analysis of joints revealed minimal cartilage destruction and inflammatory cell infiltration in anti-TNF-α-treated mice. Therefore, nanoparticle-mediated TNF-α knockdown in peritoneal macrophages may be a method to reduce local and systemic inflammation, and a novel strategy for arthritis treatment [169]. Other approach hypothesized that IL-1Ra (Interleukin-1 receptor antagonist) gene delivery can defend against inflammatory bone turnover, in rheumatoid arthritis patients. As compared to naked DNA and chitosan–DNA, folate–chitosan–DNA nanoparticles were less cytotoxic and enhanced IL-1Ra protein synthesis *in vitro*, offering a better protection against inflammation and abnormal bone metabolism *in vivo* [152].

**Table 4:** Polyethylenimine (PEI) and poly(l-lysine) (PLL)-based polymers for gene delivery.

Polymer	Therapeutic agent	Ligand	Remarks	Ref
PEG-PEI (NanoGel^TM^)	antisense oligonucleotide (ODN) targeting the mdr1 gene	transferrin insulin	Transport efficacy across the blood-brain barrier is increased by modification with transferrin or insulin. Improvement of ODN accumulation in the brain (15 fold).	[9]
lactoferrin-PEI	pDNA	lactoferrin	Selectivity for bronchial epithelial cells. Lower cellular toxicity of polyplexes and higher transfection efficiency (5-fold higher), as compared with PEI/pDNA complexes.	[170]
RGD-PEG-PEI	siRNA inhibiting vascular endothelial growth factor receptor-2	RGD	Selective tumor uptake, siRNA sequence-specific inhibition of protein expression within the tumor and inhibition of both tumor angiogenesis and growth rate.	[44]
PEI-g-PEG-RGD	pDNA (pCMV-sFlt-1)	RGD	Efficient inhibition on proliferation of endothelial cells that expressed sFlt-1 predominantly bound to exogenous VEGF and blocked the binding of VEGF to the full-length Flt-1 receptor.	[171]
siRNA-PEG-LHRH/PEI	siRNA (VEGF-vascular endothelial growth factor)	luteinizing hormone- releasing hormone (LHRH)	Enhancement of cellular uptake, as compared to those without LHRH, resulting in increased VEGF gene silencing efficiency via receptor-mediated endocytosis.	[172]
EGF-PEG-PEI	pDNA (pCMVLuc)	epidermal growth factor (EGF) peptides	EGF-containing polyplexes were 10- to 100-fold more efficient than polyplexes without EGF.	[173]
PEI	pDNA	Peptide (NL4-10K)	Polyplexes displayed no toxicity in neuronal cells. Enhancement of gene expression (up to 1000-fold) and transfection efficiency (59-fold higher), in dorsal root ganglia, compared to nontargeting polyplexes.	[174]
PEI-g-Clenbuterol	pDNA (pCMVLuc)	β_2_-adrenoceptor (clenbuterol)	Specific cellular uptake into alveolar (transfection efficiency 14-fold higher than for unmodified PEI) but not bronchial epithelial cells.	[175]
folate–PEG–PEI	pDNA (pCMV-Luc or pcDNA/rev-caspase-3)	folate	Higher transfection efficiency than other commercially available transfection agents.	[176]
PEI-PEG-Fab’	pDNA (pCMVLuc)	anti- glutamic acid decarboxylase (GAD)	Selectivity toward the islet cells. High transfection efficiency in GAD-expressing mouse insulinoma cells.	[177]
HerPEI	pDNA(pcDNA3-CMV-Luc)	anti-HER2	The HerPEI polyplexes showed significantly greater transfection activity (up to 20-folds) than nonderivatized PEI-based polyplexes in the HER2 overexpressing breast cancer cells.	[178]
mannose-PEI	pDNA	mannose	Dendritic cells transfected with polyplexes containing adenovirus particles are effective in activating T cells of T cell receptor transgenic mice in an antigen-specific fashion.	[179]
methoxypolyethyleneglycol-PEI-cholesterol	pDNA (pmIL-12)	--	Inhibition of tumor growth enhanced when combined with specific chemotherapeutic agents.	[180]
dextran-PEI	pDNA	--	Stability of the complex in the presence of BSA. The transfection efficiency depended on the molecular weight of dextran and the grafting degree.	[181]
acid-labile PEI	pDNA (pCMV-Luc)	--	The acid-labile PEI was much less toxic and showed comparable transfection efficiency to that of PEI25K. Polyplexes may be rapidly degraded in acidic endosome.	[182]
disulfide-crosslinked low molecular weight linear PEI- sodium hyaluronate	pDNA (pBR322, pEGFP-C1)	--	Polyplexes achieved significantly higher transfection efficiency than other polymer systems, especially in the presence of serum.	[183]
galactosylated PLL	pDNA (pCAT)	galactose	Hepatoma cell line revealed high gene expression. After intravenous injection, polyplexes were rapidly eliminated from the circulation and preferentially taken up by the liver’s parenchymal cells.	[184]
Lactosylated PEG-siRNA/PLL	RNAi	lactose	pH-responsive and targetable polyplexes exhibited significant gene silencing human hepatoma cells.	[185]
AWBP-PEG-PLL	pDNA (pMNK)	Artery wall binding peptide (AWBP)	High transfection efficiency in bovine aorta endothelial cells and smooth muscle cells.	[186]
Antibody-PLL	pDNA(pSV-b-galactosidase)	Anti JL1	Polyplexes internalization into Molt 4 cells and human leukemia T cells. Higher *in vitro* transfection efficiency than polyplexes without targeting ligand.	[187]
RGD-PEG-*block*-PLL	pDNA	RGD	Synergistic effect of cyclic RGD peptide and disulfide cross-links to exert the smooth release of pDNA in the intracellular environment via reductive cleavage. Enhanced transfection efficiency against HeLa cells, due to a change in their intracellular trafficking route.	[188]

Prevention of RSV (Respiratory Syncytial Virus) bronchiolitis, potentially reducing the later development of asthma associated with severe respiratory infections, was also evaluated using gene therapy. Plasmids expressing a short interfering RNA against the RSV-NS1 gene (siNS1), were complexed with chitosan. Treatment of rats with siNS1, prior to RSV exposure, was effective in reducing virus titers in the lung, preventing the inflammation and airway hyperresponsiveness associated with the infection and asthma development [189]. Similary, topical delivery of 5% imiquimod cream mixed with siRNA for natriuretic peptide receptor A (siNPRA) nanoparticles protected against asthma. In a mouse asthma model, the treatment with a imiquimod cream—containing siNPRA chitosan nanoparticles—significantly reduced the airway hyperresponsiveness, eosinophilia, lung histopathology and pro-inflammatory cytokines IL-4 and IL-5 in lung homogenates. By combining the treatment of imiquimod and siNPRA nanoparticles, a better protection against airway inflammation was achieved [190].

The feasibility of applying nanotechnology against *Plasmodium falciparum*, malarial parasites, was also investigated using antisense ODNs (phosphorothioate antisense oligodeoxynucleotides) against malarial topoisomerase II gene, incorporated in chitosan nanoparticles. The *in vivo* use of nanoparticles against malaria is possible only if they are harmless to red blood cells. The erythrocyte membrane contains anionic glycoproteins, which can interact with protonated amino groups of chitosan. This process induces a curvature of the cell membrane, leading to rupture and hemoglobin release. In the presence of plasma, however, the membrane damage is reduced, an effect that might be explained by adsorption of negatively charged plasma proteins on the surface of charged particles; the plasma shielding effect suggests that chitosan nanoparticles indeed do not harm erythrocytes *in vivo*. Nanoparticles with negative surface charge exhibited a significantly stronger inhibitory effect (∼87% inhibition) on the parasite growth in comparison to the positive ones (∼74% inhibition) or free ODNs (∼68% inhibition). This was the first study demonstrating the susceptibility of human malaria parasite to antisense nanoparticles [146].

**Table 5:** Other synthetic polymers for gene delivery.

Polymer	Therapeutic agent	Remarks	Ref
poly(imidazole/ 2-dimethylaminoethylamino)phosphazene	pDNA	Imidazole effect on cytotoxicity and transfection efficiency. Evaluation of half-lives under neutral and acidic conditions.	[144]
poly[α-(4-aminobutyl)-L-glycolic acid] (PAGA)	pDNA( pCAGGS-Il10, pCAGGS-Il4)	Combined administration of mouse *Il4* and *Il10* plasmids prevents the development of autoimmune diabetes in non-obese diabetic mice.	[191]
poly(4-hydroxy-L-proline ester)	pDNA(CMV-*β*Gal)	The minimum viability of cells incubated with poly(4-hydroxy-L-proline ester) was 85%, which is excellent when compared to the cases of polylysine (20%) and polyethylenimine (2%).	[192]
poly(amido amine)s containing multiple disulfide linkages	pDNA	Buffer capacity of poly(amido amine)s in the pH range 7.4-5.1. High transfection efficiency and gene expression, in the presence of serum.	[193]
cationic amphoteric polyamidoamine	pDNA (pEGFP)	Evaluation of toxicity and hemolytic activity in the pH range 4.0-7.4. Circulation time and organ accumulation assessment. Study of complex stability and transfection efficiency	[194]
three blocks of amino acids Ac-(AF)6-H5-K15-NH2 (FA32)	Doxorubicin, pCMV-luciferase, pCMV-p53	Co-delivery of drug and gene using nanoparticles was demonstrated via confocal imaging, luciferase expression in the presence of doxorubicin, and synergy in cytotoxic effect towards HepG2 cells.	[166]
*N*,*N*-diethylethylenediamine-polyurethane	pDNA (pCMV-*β*gal)	Cytotoxicity was substantially lower and transfection efficiency comparable to the well-known gene carrier poly(2-dimethylaminoethyl methacrylate	[195]

## 5. Conclusion

Nanoparticulate systems are new tools that promise a revolution in the field of drug delivery. Nanodevices are suited to achieve the ideal of a controlled and targeted release of bioactive molecules. Among the available nanosystems, self-assembled polymeric nanogels are particularly attractive, since they are easy to produce, affordable, and may effectively incorporate a variety of drugs, including biopharmaceuticals. Furthermore, they may be decorated with different kinds of molecules, improving the stability and target ability.

However, the development of these systems, consisting either of amphiphilic molecules or electrostatically stabilized macromolecular complexes, still require a more comprehensive characterization before their full potential can be exploited. The molecular organization of the nanogels, nanotoxicology, interaction with cells and tissues, including the *in vivo* biodistribution and intracellular trafficking, are among the issues deserving a more comprehensive characterization.
